# Characterization of the complete chloroplast genome of *Heliopsis helianthoides* (Asteraceae) and its phylogenetic position, representing the first in the genus *Heliopsis*

**DOI:** 10.1080/23802359.2026.2742730

**Published:** 2026-10-04

**Authors:** Shu Wang, Anqi Du, Mingyu Mei, Chaojun Chen, Yanuo Liu, Liqiang Wang

**Affiliations:** College of Pharmacy, Heze University, Heze, Shandong Province, P. R. China

**Keywords:** Asteraceae, chloroplast genome, Heliopsis helianthoides, phylogenetics

## Abstract

*Heliopsis helianthoides* is a perennial medicinal plant in the Asteraceae family, valued for its anti-inflammatory, antimicrobial, and antioxidant properties. Despite its pharmacological significance, its chloroplast genome remains uncharacterized. Here, we report the first complete chloroplast genome of *H. helianthoides*, which is also the first for the genus *Heliopsis*. The genome is 151,140 bp in length with a typical quadripartite structure, containing 127 genes and a GC content of 37.6%. Phylogenetic analysis revealed that *H. helianthoides* clusters with *Acmella*, *Echinacea*, and *Zinnia* within the subtribe Zinniinae (Heliantheae). This genome provides valuable resources for future evolutionary and taxonomic studies of *Heliopsis*.

## Introduction

*Heliopsis helianthoides* (Linnaeus) Sweet (1826), commonly known as smooth oxeye, is a perennial Asteraceae species, native to North America with diverse medicinal properties and bioactive potential. It bears erect stems and lanceolate leaves, and contains diverse bioactive compounds including N-alkylamides, lignans (Hajdu et al. 2014a, 2014b), phenolic acids and flavonoids such as chlorogenic acid, rutin, luteolin, and apigenin-7-glucoside (Pavlenko-Badnaoui et al. 2021). These constituents underlie its anti-inflammatory, antimicrobial, and antioxidant activities (Hajdu et al. 2014a, 2014b; Pavlenko-Badnaoui et al. 2021), making it a candidate for natural product and therapeutic research.

*Heliopsis helianthoides* belongs to Heliantheae (Asteraceae). *Heliopsis*, characterized by persistent phyllaries and distinctive achenes, has been recovered as monophyletic in molecular phylogenetic analyses (Schilling and Panero 2002). Chloroplast DNA (*ndh*F, *trn*L-F) and nuclear ITS data place *Heliopsis* as a distinct genus closely related to *Helianthus* within Heliantheae (Panero and Funk 2008). Phylogenetic reconstructions suggest within-genus diversification, but relationships among infraspecific taxa remain unresolved (Urbatsch et al. 2000). A species-level phylogeny integrating population genetics is still needed to clarify the biogeographic history of *H. helianthoides* (Mandel et al. 2019).

While the phylogenetic position of *H. helianthoides* has been increasingly clarified, Asteraceae chloroplast genomes reveal conserved quadripartite structures with lineage-specific IR variations (Nguyen et al. 2024), and similar studies have identified hypervariable regions useful for species discrimination (Akrami et al. 2025; Hejazi et al. 2025; Soorni and Golchini 2025). Single-species chloroplast genome report has also provided resources for understudied genera (Huynh et al. 2024). However, no chloroplast genome has been reported for *Heliopsis*. Herein, we assembled the first complete chloroplast genome of this species, providing foundational resources for evolutionary and genetic studies, and facilitating future investigations into population structure, phylogeography, and germplasm characterization of *Heliopsis* and allied genera.

## Materials and methods

### Plant materials and DNA extraction

*Heliopsis helianthoides* samples (Figure 1) were collected on the campus of Heze University, Shandong, China. A voucher specimen was deposited in the Herbarium of Heze University (voucher ID: HZU-2025-0803; contact: Liqiang Wang, lys832000@163.com; www.hezeu.edu.cn). Total genomic DNA was isolated from healthy fresh leaves using the Tiangen Plant Genomic DNA Kit (Tiangen Biotech (Beijing) Co., Ltd.). DNA quality and integrity were assessed *via* 1% agarose gel electrophoresis and NanoDrop spectrophotometry prior to sequencing.

**Figure 1.**
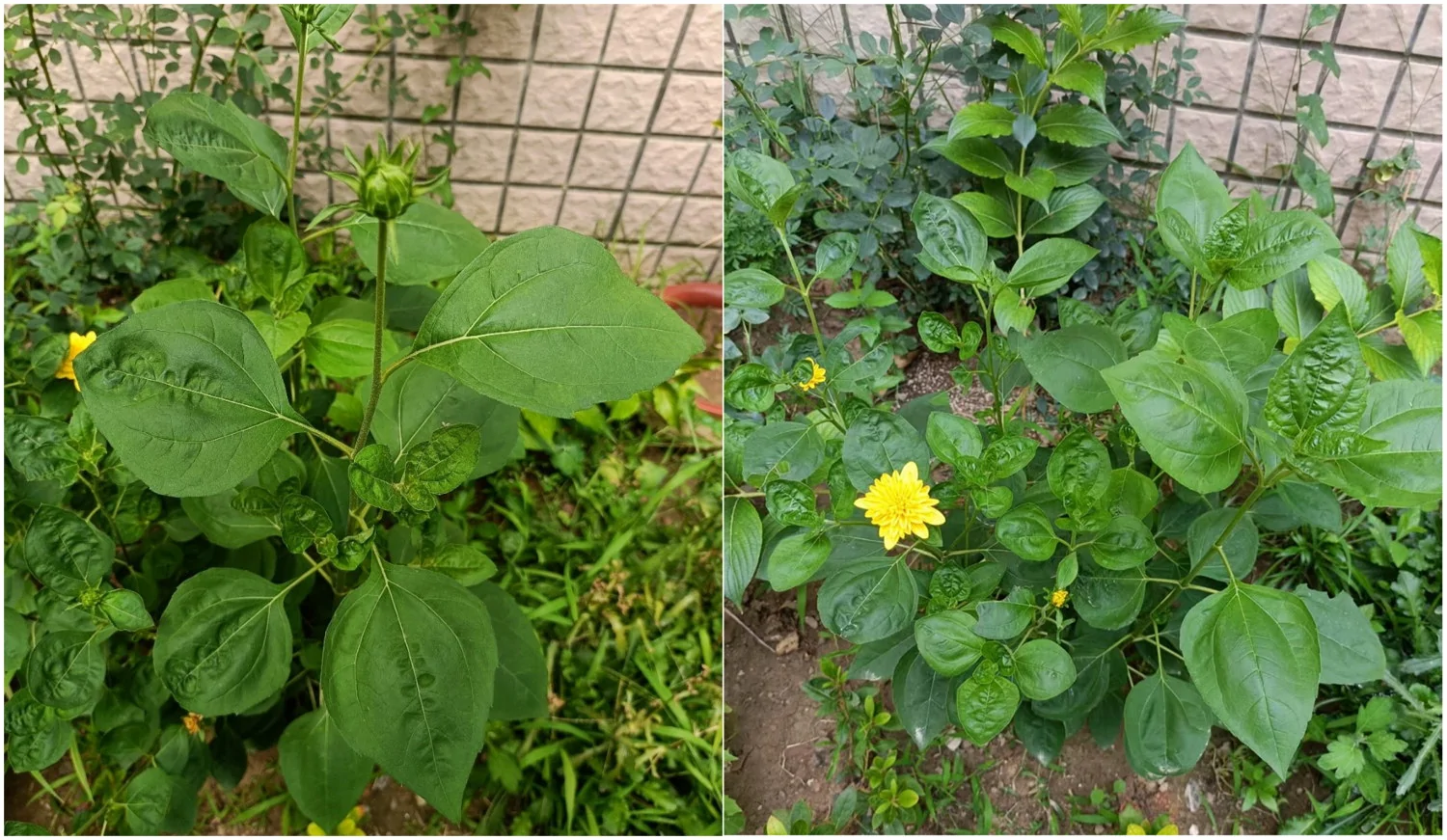
Field photographs of *Heliopsis helianthoides* showing habit and diagnostic features (photographer: Liqiang Wang). Perennial herbs, 40–150 cm tall. Stems glabrous or slightly hispid. Leaves ovate-lanceolate, margin serrate; upper surface glabrous to scabrous, lower surface pubescent. Capitulum terminal, solitary or rarely paired; ray florets bright yellow, apices acute-acuminate. Achenes dark brown, 4-angled.

### Genome sequencing, assembly and annotation

Qualified DNA was fragmented for library construction using the Hieff NGS^®^ OnePot Pro DNA Library Prep Kit V4 (YEASEN, Shanghai, China), with genomic DNA enzymatically sheared to 200–400 bp. Paired-end (150 bp) high-throughput sequencing was performed on the DNBSEQ platform (Benagen Tech, Wuhan, China). Raw reads were filtered in Fastp v0.21.0 (Chen et al. 2018) to remove reads with >5% N bases, ≥50% low-quality bases (*Q* ≤ 5), adapters and PCR duplicates. High-quality reads were assembled *de novo* using GetOrganelle (v1.7.6.1) (Jin et al. 2020). The assembled genome was annotated using CPGAVAS2 (Shi et al. 2019), followed by manual curation.

### Phylogenetic analysis

To resolve the phylogenetic position of *H. helianthoides*, we downloaded chloroplast genomes for 14 Zinniinae species from four genera (*Echinacea*, *Rudbeckia*, *Silphium*, *Zinnia*) and 5 Ecliptinae species from three genera (*Acmella*, *Eclipta*, *Sphagneticola*) (Skibicki 2025). Distant genera (*Aldama*, *Verbesina*, *Helianthus*, *Xanthium*) were excluded. For taxa with multiple chloroplast genomes, one was randomly selected. *Inula grandis* and *Inula salicina* served as outgroups, consistent with prior Asteraceae phylogenies (Panero and Funk 2008), as Inuleae is sister to Heliantheae. Seventy-three shared protein-coding genes (PCGs) were extracted from the 22 chloroplast genomes. PCGs were aligned with MAFFT (v7.505; Katoh and Standley 2013) and concatenated. A maximum-likelihood (ML) tree was built in IQ-TREE (v2.2.2.7) (Nguyen et al. 2015) under TVM + F + I + R5 model, with 1000 bootstrap replicates.

### RSCU and Pi analysis

Relative Synonymous Codon Usage (RSCU) was calculated in PhyloSuite (v2) (Zhao et al. 2025) and visualized *via* the RSCU-Plot Shiny app (https://pcg-lab.shinyapps.io/RSCU-Plot/) following Diani Gohar and Soorni (2025). Nucleotide diversity (Pi) was estimated using CPStools (Huang et al. 2024). Ingroup taxa was selected for these analyses.

## Results

Approximate 26 GB raw fastq data were generated for the first chloroplast genome assembly of *H. helianthoides*. The assembled complete chloroplast genome is 151,140 bp in length with a GC content of 37.6%. It contains a pair of inverted repeats (IRa and IRb) of 24,622 bp, separated by a large single-copy region (LSC) of 83,601 bp, and a small single-copy region (SSC) of 18,295 bp (Figure 2). The genome achieved an average sequencing depth of 315.45 ×, ranging from 52× to 3,512× (Figure S1).

**Figure 2.**
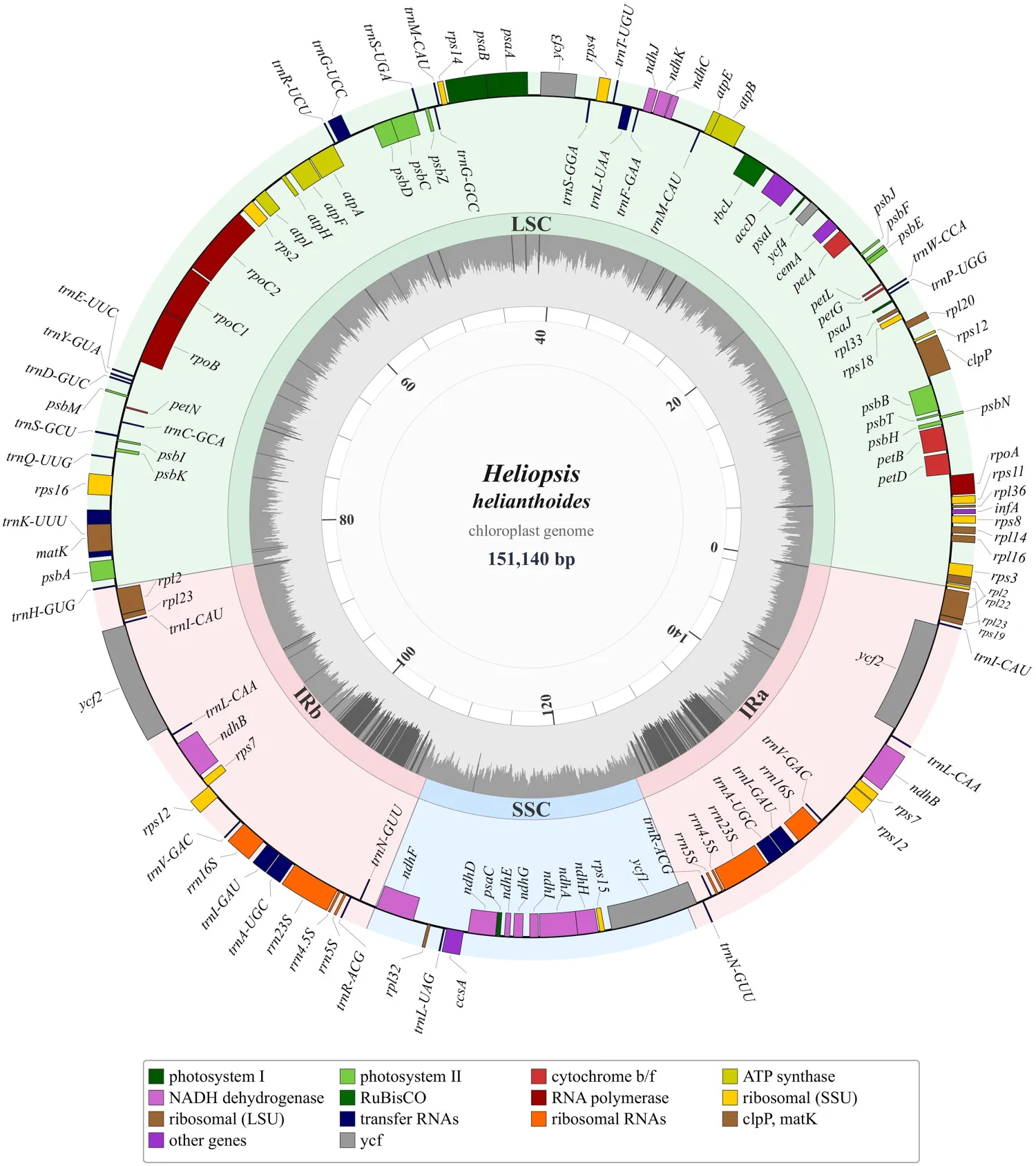
The chloroplast genome map of *Heliopsis helianthoides*. The chloroplast genome displayed the typical quadripartite structure consisting of a large single-copy (LSC) region, a small single-copy (SSC) region, and a pair of inverted repeats (IRa and IRb). Genes were color-coded by functional category and were distributed on both strands of the genome. Functional categories included genes involved in photosystem I and II, cytochrome b6/f complex, ATP synthase, NADH dehydrogenase, RNA polymerase, ribosomal proteins (small and large subunits), Rubisco, tRNAs, rRNAs, and hypothetical chloroplast reading frames (*ycf*).

A total of 127 genes (109 unique genes) were annotated, including 84 PCGs (78 unique), 35 tRNA genes (27 unique), and 8 rRNA genes (4 unique). Genes, such as *atp*F, *ndh*A, *ndh*B (×2), *pet*B, *pet*D, *rpl*2 (×2), *rpo*A, *rpo*C1, *rps*16, *trn*A-UGC (×2), *trn*G-UCC, *trn*I-GAU (×2), *trn*K-UUU, and *trn*L-UAA contain one intron (Figure S2, Table S1). Genes such as *clp*P, and *ycf*3 harbor two introns (Figure S2, Table S1). The *rps*12 is trans-spliced: the 5′ exon is in the LSC, while the duplicated 3′ exons are in IRa and IRb, each containing one intron (Figure S3, Table S1), consistent with other Asteraceae and Myrtaceae chloroplast genomes (Huynh et al. 2024; Nguyen et al. 2024).

The ML tree showed high bootstrap support across all nodes. All genera formed monophyletic clades, and *H. helianthoides* clustered with *Acmella*, *Echinacea* and *Zinnia* species (Figure 3). This robust phylogeny confirms the taxonomic placement of *H. helianthoides* within Heliantheae. To further characterize the *H. helianthoides* chloroplast genome, RSCU and Pi analyses were performed accross seven Heliantheae species: *H. helianthoides*, *Acmella paniculata*, *Zinnia elegans*, *Echinacea angustifolia*, *Echinacea purpurea*, *Rudbeckia hirta*, and *Silphium integrifolium*. RSCU analysis revealed a strong preference for A/U-terminated codons in all seven taxa, with GCU (Ala), UUA (Leu), and UCU (Ser) showing the highest values (RSCU > 1.75; Figure S4). Pi analysis identified the gene *ycf*1 (Pi = 0.02918) and intergenic spacer *atp*F_2-*atp*A (Pi = 0.07381) as highly variable regions (Figure S5), suggesting potential DNA barcodes.

**Figure 3.**
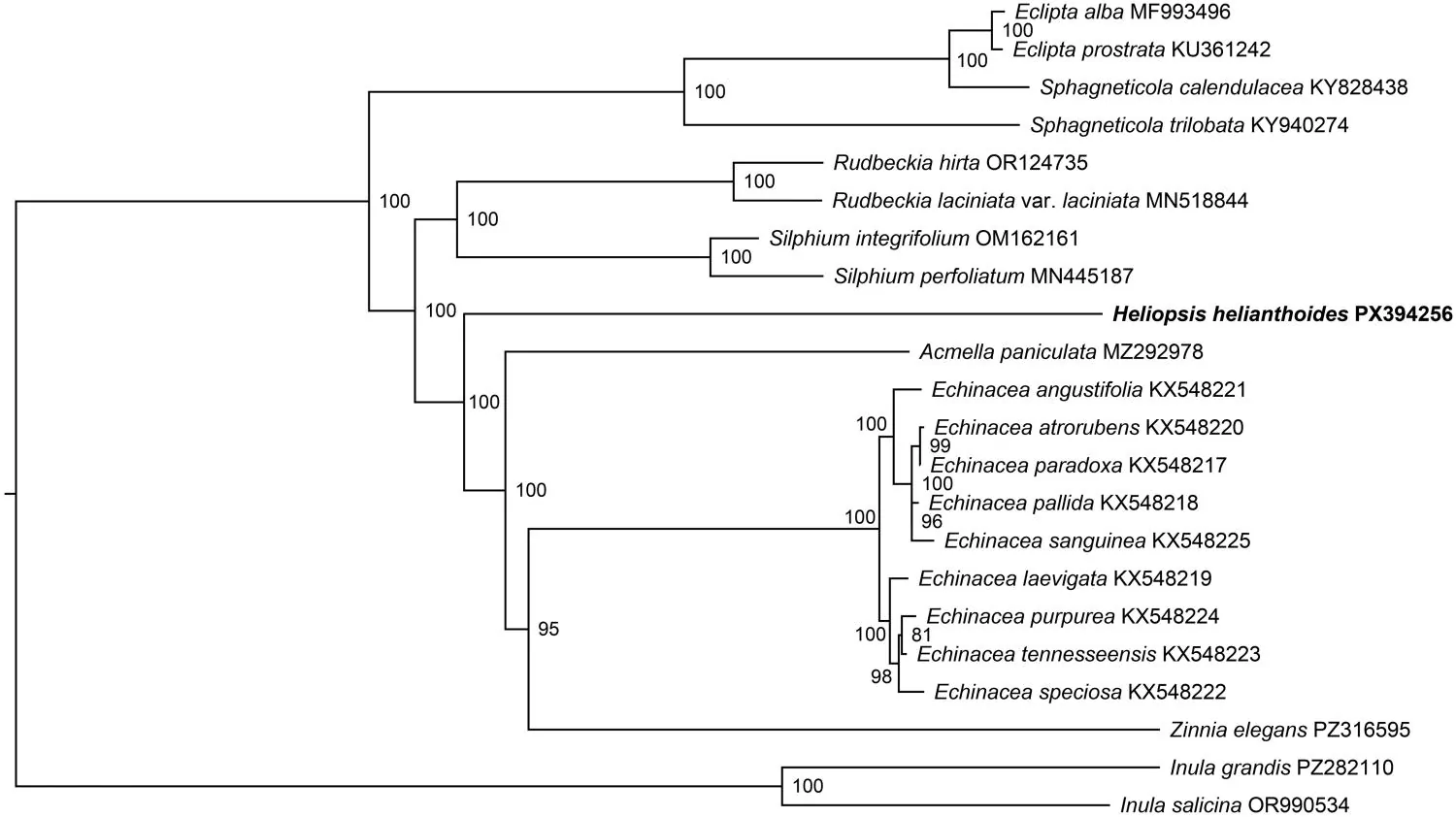
ML phylogeny of *Heliopsis helianthoides* and its close relatives. The bootstrap values based on 1000 replicates were shown on each node in the phylogenetic tree. Twenty-one species downloaded from GenBank plus *H. helianthoides* (PX394256, labeled by bold font) reported in this study: *Inula grandis* (outgroup, PZ282110) *Inula salicina* (outgroup, OR990534), *Eclipta alba* MF993496 (Kim et al. 2017), *Eclipta prostrata* KU361242 (Park et al. 2016), *Sphagneticola calendulacea* KY828438 (Ng et al. 2018), *Sphagneticola trilobata* KY940274 (Zhou et al. 2018), *Rudbeckia hirta* OR124735, *Rudbeckia laciniata* var. *laciniata* MN518844, *Silphium integrifolium* OM162161, *Silphium perfoliatum* MN445187, *Acmella paniculata* MZ292978, *Echinacea paradoxa* KX548217 (Zhang et al. 2017), *Echinacea pallida* KX548218 (Zhang et al. 2017), *Echinacea laevigata* KX548219 (Zhang et al. 2017), *Echinacea atrorubens* KX548220 (Zhang et al. 2017), *Echinacea angustifolia* KX548221 (Zhang et al. 2017), *Echinacea speciosa* KX548222 (Zhang et al. 2017), *Echinacea tennesseensis* KX548223 (Zhang et al. 2017), *Echinacea purpurea* KX548224 (Zhang et al. 2017), *Echinacea sanguinea* KX548225 (Zhang et al. 2017), and *Zinnia elegans* PZ316595.

## Discussion and conclusion

Herein, we report the first chloroplast genome of *H. helianthoides*. The genome is 151,140 bp in length with a typical quadripartite structure. It contains 127 genes, and has a GC content of 37.6%, consistent with the conserved quadripartite structure reported across other Asteraceae plastomes (Nguyen et al. 2024). In genome length, gene content, and GC content, the genome closely resembles those of *Acmella paniculata*, *Zinnia elegans*, and *Echinacea* species (Zhang et al. 2017), clustering together as a monophyletic group in the ML tree.

Phylogenetic analysis based on 73 shared PCGs (Figure 3) clearly resolved the systematic position of *H. helianthoides* within the subtribe Zinniinae (Heliantheae). The topology revealed that *H. helianthoides* clusters with *Acmella*, *Echinacea*, and *Zinnia*, forming the core clade of Zinniinae. This result is highly congruent with previous phylogenomic studies based on chloroplast genome and nuclear data (Zhang et al. 2017; Skibicki 2025), confirming Zinniinae as a distinct and stable monophyletic lineage within Heliantheae. Morphological evidence also supports this relationship, as the three genera share conical or subconical receptacles and certain cypselar features (Fisher 1957; McGregor 1968).

RSCU analysis revealed strong for A/U-ending codon preference in the *H. helianthoides* chloroplast genome, consistent with the general pattern reported in other Asteraceae and Lamiaceae plastomes (Akrami et al. 2025; Hejazi et al. 2025; Soorni and Golchini 2025). Pi analysis identified *ycf*1 and *atp*F_2-*atp*A as highly variable regions. *ycf*1 has been widely recognized as a candidate barcode (Huynh et al. 2024; Nguyen et al. 2024), while *atp*F_2-*atp*A represents a novel hypervariable region. These loci serve as valuable markers for future species identification and population-level studies within *Heliopsis* and allied genera.

In summary, the chloroplast genome of *H. helianthoides* is structurally conserved within Zinniinae (Heliantheae), and our phylogenetic analysis supports its placement in this subtribe. The new genomic resource facilitates future studies on evolutionary history, infraspecific relationships, and broader taxonomy of Heliantheae.

## Supplementary Material

Supplymentary_Figures.docx

Table_S1.docx

## Data Availability

The complete chloroplast genome sequence of *H. helianthoides* in this study has been submitted to the NCBI database (https://www.ncbi.nlm.nih.gov) under the accession number PX394256. The associated BioProject, BioSample, and SRA numbers are PRJNA1146888, SAMN62231412, and SRR40038071.
