# Supplementary material for: Characterization of the complete chloroplast genome of Heliopsis helianthoides (Asteraceae) and its phylogenetic position, representing the first in the genus Heliopsis
Source: Mitochondrial DNA B Resour. 2026 Oct 4;11(11):1261–5. doi: 10.1080/23802359.2026.2742730 (PMC13644442; doi:10.1080/23802359.2026.2742730)
Supplement: Supplementary file 2 [file TMDN_A_2742730_SM4278.docx]

**Figure S1. Sequencing depth and coverage maps validated the high-quality assembly of the *Heliopsis helianthoides* chloroplast genome.** Depth plots were generated using DrawSeqDepth (https://github.com/wlqg1983/DrawSeqDepth) with default parameters. The default parameters included mapping software minimap2. The sequencing depth was calculated by samtools.

**Figure S2. Schematic map of the cis-spliced protein-coding genes in the chloroplast genome of *Heliopsis helianthoides*.** Gene names were shown on the left, gene structures on the right. Exons are represented in black, introns in white. Arrows indicated the direction of the sense strand. Exon and intron lengths were not drawn to scale.

**Figure S3. Map of the trans-spliced gene (*rps*12) of *Heliopsis helianthoides*.** Exons were shown in black and grey, introns in white. Arrows indicated the direction of the sense strand.

**Figure S4. Relative synonymous codon usage (RSCU) of the chloroplast genome of *Heliopsis helianthoides* and six related Zinniinae species.** The x-axis represents 20 amino acids, while the height of the bar chart indicates the number of codons corresponding to each amino acid.

**Figure S5. Polymorphism across chloroplast regions of Heliopsis helianthoides and six related Zinniinae species.** Black circles indicate ten highly polymorphic loci. The x-axis shows chloroplast regions, divided into large single-copy (LSC), small single-copy (SSC), and inverted repeat (IR) regions; the y-axis shows nucleotide diversity (π). (A) Coding genes. (B) Intergenic spacer (IGS) regions.
